# Predictive Value of Machine Learning for Platinum Chemotherapy Responses in Ovarian Cancer: Systematic Review and Meta-Analysis

**DOI:** 10.2196/48527

**Published:** 2024-01-22

**Authors:** Qingyi Wang, Zhuo Chang, Xiaofang Liu, Yunrui Wang, Chuwen Feng, Yunlu Ping, Xiaoling Feng

**Affiliations:** 1 Department of First Clinical Medical College Heilongjiang University of Chinese Medicine Harbin China; 2 Basic Medical College Heilongjiang University of Chinese Medicine Harbin China; 3 Department of Gynecology First Affiliated Hospital of Heilongjiang University of Chinese Medicine Harbin China

**Keywords:** ovarian cancer, platinum chemotherapy response, machine learning, platinum-based therapy, predictive potential

## Abstract

**Background:**

Machine learning is a potentially effective method for predicting the response to platinum-based treatment for ovarian cancer. However, the predictive performance of various machine learning methods and variables is still a matter of controversy and debate.

**Objective:**

This study aims to systematically review relevant literature on the predictive value of machine learning for platinum-based chemotherapy responses in patients with ovarian cancer.

**Methods:**

Following the PRISMA (Preferred Reporting Items for Systematic Reviews and Meta-Analyses) guidelines, we systematically searched the PubMed, Embase, Web of Science, and Cochrane databases for relevant studies on predictive models for platinum-based therapies for the treatment of ovarian cancer published before April 26, 2023. The Prediction Model Risk of Bias Assessment tool was used to evaluate the risk of bias in the included articles. Concordance index (C-index), sensitivity, and specificity were used to evaluate the performance of the prediction models to investigate the predictive value of machine learning for platinum chemotherapy responses in patients with ovarian cancer.

**Results:**

A total of 1749 articles were examined, and 19 of them involving 39 models were eligible for this study. The most commonly used modeling methods were logistic regression (16/39, 41%), Extreme Gradient Boosting (4/39, 10%), and support vector machine (4/39, 10%). The training cohort reported C-index in 39 predictive models, with a pooled value of 0.806; the validation cohort reported C-index in 12 predictive models, with a pooled value of 0.831. Support vector machine performed well in both the training and validation cohorts, with a C-index of 0.942 and 0.879, respectively. The pooled sensitivity was 0.890, and the pooled specificity was 0.790 in the training cohort.

**Conclusions:**

Machine learning can effectively predict how patients with ovarian cancer respond to platinum-based chemotherapy and may provide a reference for the development or updating of subsequent scoring systems.

## Introduction

### Background

Ovarian cancer is one of the most common cancers worldwide [1], and this gynecological cancer is characterized by poor prognosis and high mortality [2]. It is estimated that epithelial ovarian cancer (EOC) represents 90% of the ovarian cancer cases [3], with serous carcinoma being the most common pathological type [4]. Because of the absence of cancer-specific symptoms and effective screening techniques, EOC is frequently diagnosed at a late stage [5,6]. Despite undergoing relevant treatments, patients with ovarian cancer still face high rates of recurrence and mortality, with a 5-year survival rate of <30% [7]. According to GLOBOCAN 2020, the number of new ovarian cancer cases in low and high Human Development Index countries will increase by approximately 96% and 19%, respectively, by 2040 [8].

Currently, the National Comprehensive Cancer Network Guidelines (2023 Edition) recommend the use of paclitaxel or carboplatin for 3 weeks as the first-line treatment for stage 2 to 4 EOC [9]. Although platinum-based chemotherapy is effective in most patients with ovarian cancer, resistance may occur in some patients [10]. In addition, their response to platinum treatment is unknown until chemotherapy is completed. The platinum-free interval is a reliable indicator for predicting treatment efficacy and patient prognosis because it can evaluate whether patients with ovarian cancer respond to platinum drugs and their recurrence [11,12]. The Gynecologic Cancer Group divides responses to platinum chemotherapy into 4 categories based on the duration of platinum-free interval (platinum refractory: <1 mo, platinum-resistant: 1-6 mo, partial platinum response: 6-12 mo, and platinum response: >12 mo) [13]. The chemoresistance of ovarian cancer may be related to genome expression [14,15], tumor heterogeneity, intestinal microbiota, DNA repair [16], DNA methylation [17,18], and mitochondrial function [19,20] related to immunoediting. Within 2 years, approximately 70% of these patients relapse [21]. Therefore, predicting the response to platinum-based chemotherapy in patients with ovarian cancer is critical. Despite the emergence of multiple approaches, including mutational signatures, transcriptomic signatures, tumor mutation burden, and functional biomarkers, there is no conclusive evidence to guide the precise treatment of patients with ovarian cancer [22].

### Objectives

In recent years, with the increasing availability of medical data and the continuous improvement in computer analysis capabilities, machine learning has been increasingly used in the medical field [23,24]. Machine learning is a technological application that uses algorithms and data to enable computers to automatically learn and enhance. It excels in handling large amounts of complex and multidimensional information, thereby improving the accuracy and efficiency of decision-making [25-27]. In various domains of oncology, machine learning has been used to identify cancer imaging features [28], predict the risk of cancer recurrence [29], screen cancer drug targets [30], and optimize cancer treatment options [31]. Some researchers have explored machine learning–based methods to construct prediction models for platinum reactions in ovarian cancer. However, in the field of machine learning research, there is a diverse range of methods and variables. The predictive performance of these methods for outcome events remains controversial. Currently, in evidence-based medicine, a comprehensive summary of the predictive performance of machine learning is lacking. Therefore, we conducted this study to explore early risk stratification in response to platinum-based chemotherapy in patients with ovarian cancer. Our aim was to enhance chemotherapy management in patients with ovarian cancer.

## Methods

This study was carried out following the PRISMA (Preferred Reporting Items for Systematic Reviews and Meta-Analyses) 2020 guidelines (Table S1 in Multimedia Appendix 1) and registered on PROSPERO (CRD42022340928).

### Data Sources and Searches

Relevant studies published before March 15, 2022, were thoroughly searched in the PubMed, Web of Science, Embase, and Cochrane databases. Search terms included subject headings (Medical Subject Headings in PubMed and Emtree in Embase) and free words, such as “Ovarian Neoplasms,” “machine learning,” “prediction model,” and “Platinum-Based Chemotherapy.” The specific search strategy is presented in Table S1 in Multimedia Appendix 2. To prevent the risk of missing new literature, we conducted a supplementary search of each database until April 26, 2023.

### Inclusion and Exclusion Criteria

The inclusion criteria were as follows:

- Patients diagnosed with ovarian cancer
- The research types are case-control, cohort, nested case-control, and case cohort studies.\
- A completely constructed predictive model for platinum chemotherapy (platinum-sensitive or platinum-resistant) response in patients with ovarian cancer
- Studies without external validation
- Different machine learning studies published on the same data set
- The literature written in English

Meanwhile, the following studies were excluded:

- The research type was meta-analysis, review, guideline, expert opinion, etc
- Only a risk factor analysis was carried out, but no complete machine learning prediction model was developed
- The following outcome measures were used: receiver operating characteristic curve, concordance index (C-index), sensitivity, specificity, accuracy, recovery rate, precision rate, confusion matrix, diagnostic 4-grid table, *F*_1_-score, and calibration curve. The original study should include at least one of the above indicators. Missing studies need to be excluded.
- Studies with small sample sizes (<50 cases)
- Research on the accuracy of single-factor prediction

### Literature Screening and Data Extraction

The retrieved studies were imported into EndNote (Clarivate Plc) to remove duplicate publications automatically and manually. Subsequently, we reviewed the titles or abstracts of the remaining studies to exclude original studies that did not align with the topic. We proceeded to read the full texts of the remaining studies to screen the original studies that ultimately met the criteria.

The following information was collected from each eligible study: first author, year of publication, location, research duration, population characteristics, background, number of hospitals, study design (prospective or retrospective), number of patients, age, histological classification of enrolled patients, presence of tumor deposit after treatment, treatment methods, prediction objects, chemotherapy methods, number of positive samples, number of training set samples, total number of samples, follow-up time, variable selection method, characteristics of the machine learning approach (specific algorithm or type), validation method (cross-validation, retention method, external validation, or none), number of model variables, included model variables, efficacy evaluation indicators, sample size of the validation set, and prediction results.

The literature screening and data extraction were independently conducted by 2 researchers (QW and ZC). Following completion, crosschecks were performed. In the event of any disputes, a third researcher (XL) was consulted to assist in resolution.

### Quality Assessment

The Prediction Model Risk of Bias Assessment (PROBAST), a technique for predicting the model risk of bias, was used to assess the risk of bias in the predictive models reported in eligible studies [32,33]. This tool consists of 4 major domains, participants, predictors, outcomes, and statistical analysis, and it reveals the overall risk of bias and applicability. The 4 domains have 2, 3, 6, and 9 distinct questions, respectively, with 3 possible answers: yes or probably yes, no or probably no, and no information. A domain is deemed high risk if it receives at least 1 no or probably no question, whereas a domain that receives yes or probably yes for all of its questions is considered low risk. When all domains are classified as low risk, the overall risk of bias is graded as low. Meanwhile, when at least one domain is deemed high risk, the overall risk of bias is regarded as high. Two investigators independently assessed the risk of bias and crosschecked their findings using PROBAST. Any disagreements were resolved by discussion with a third researcher. The assessment of the risk of bias was independently conducted by 2 researchers (YW and CF). Upon completion, a crosscheck was performed. In case of any dispute, a third researcher (YP) was consulted to assist in the decision-making process.

### Data Analysis

If the C-index lacked a 95% CI and SE, we referred to the study by Debray et al [34], which estimated its SEs. Because machine learning encompasses a wide range of mathematical models and predictive factors, there is high heterogeneity among various studies. Hence, a random effects model was used for the meta-analysis. In addition, we used a bivariate mixed effects model, which is a random effects model, to perform the meta-analysis of sensitivity and specificity. At the same time, we used the heterogeneity index (*I*^2^) to measure the heterogeneity. *P*<.05 indicated a statistically significant difference. Moreover, subgroup analysis was conducted to increase the robustness of the results and reduce heterogeneity between studies, according to the different types of prediction models and the possible influencing factors, for instance, whether it is high-grade serous ovarian cancer and whether there is a tumor deposit.

### Ethical Considerations

All analyses were based on previously published studies; therefore, ethics approval and patient consent were not required.

## Results

### Search Strategy

**Figure 1:**
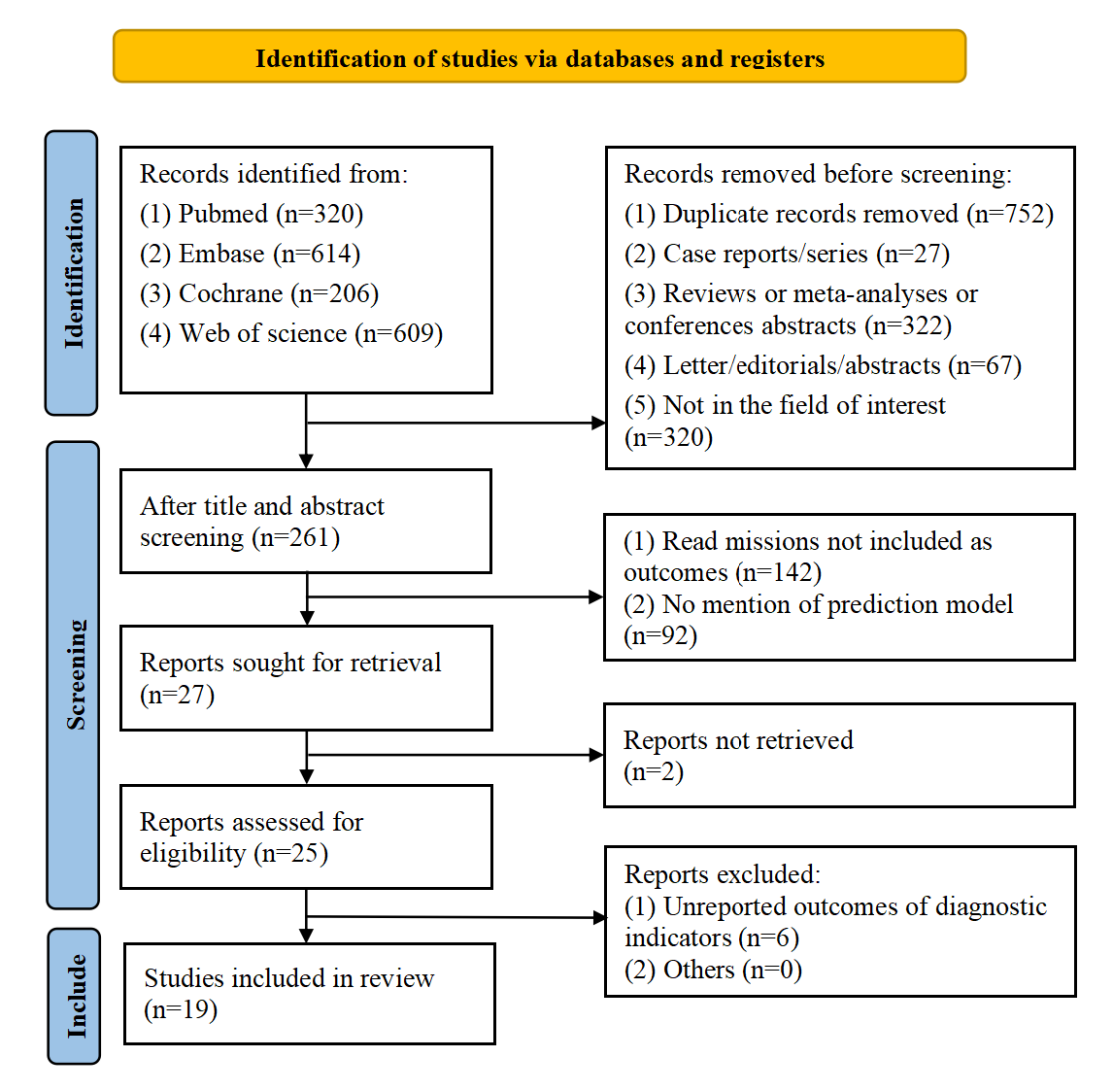
Flow diagram of the study selection process.

A total of 1749 articles were obtained from the PubMed, Web of Science, Embase, and Cochrane databases. After removing 752 duplicates, we screened the titles and abstracts and identified 261 potentially eligible articles. On the basis of a full-text review, 242 studies were excluded, with 234 (96.7%) studies deleted for inappropriate outcomes, 6 (2.5%) studies deleted for inadequate data, and 2 (0.8%) studies deleted for no access to the full text. Finally, this study included 19 articles. Figure 1 shows the study search strategy.

### Characteristics of Included Studies

The basic characteristics of the 19 eligible articles [35-53] are presented in Table 1. Of the 19 studies, only one consisted of patients who had recurrent ovarian cancer, whereas the remaining 18 studies involved patients who had primary ovarian cancer. There were 3 multicenter studies, 5 single-center studies, and 11 database-based studies. In total, 15 studies were externally validated. The 19 eligible studies involved 7137 patients, and the number of patients included ranged from 58 to 1002. These eligible studies contained 39 predictive models, of which 22 reported sensitivity and specificity. The most widely used modeling methods in the training cohort were logistic regression (LR; 16/39, 41%), Extreme Gradient Boosting (XGBoost; 4/39, 10%), and support vector machines (SVMs; 4/39, 10%), whereas the common modeling method in the validation cohort was SVM (4/12, 33%).

**Table 1:** Characteristics of the included studies.

Study, year	Country	Sample source	Chemotherapy	Positive samples, n/N (%)	Training set samples, n/N (%)	Overall sample size, n	Variable selection method	Type of model	External validation	External validation sample size
Shannon et al [35], 2021	Singapore	GDSC^a^, TCGA^b^, and GEO^c^	Carboplatin	39/50 (78)	50/60 (83)	60	—^d^	SVM^e^, LR^f^, KNN^g^, DT^h^, AdaBoost^i^, GBM^j^, and XGBoost^k^	1	10
Hwangbo et al [37], 2021	Korea	The Seoul National University Hospital, Asan Medical Center, and Severance Hospital	Platinum-based combination chemotherapy	779/1002 (77.7)	1002/1002 (100)	1002	Univariate and multivariate analysis	LR, RF^l^, SVM, and DNN^m^	0	—
Zhao et al [38], 2019	China	GEO and TCGA	Platinum-based combination chemotherapy	42/129 (32)	129/707 (18.2)	707	Univariate and multivariate analysis	LR, COX^n^, SVM, and ANN^o^	1	454
Paik et al [39], 2017	Korea	Samsung Medical Center	Platinum-based combination chemotherapy	616/757 (81.4)	757/757 (100)	757	Univariate and multivariate analysis	LR	0	—
Han et al [40], 2012	China	TCGA and GEO	Platinum or paclitaxel-based treatment	177/200 (88.5)	200/322 (62.1)	322	Principal components method	SPC^p^	1	122
Lan et al [36], 2019	China	Sun Yat-Sen University Cancer Center	Platinum or paclitaxel-based treatment	22/71 (31)	71/91 (78)	91	Univariate and multivariate analysis	LR	0	—
Zheng et al [41], 2021	China	Beijing Cancer Hospital, Peking Union Medical College and TCGA	Taxol plus platinum-based chemotherapy	44/60 (73)	60/448 (13)	448	Univariate and multivariate analysis	COX	1	388
Yi et al [42], 2021	China	Hunan Cancer Hospital	Platinum-based combination chemotherapy	26/71 (36)	71/102 (69)	102	LASSO^q^	RF and SVM	1	31
Yu et al [43], 2020	America	TCGA and CPTAC^r^	Platinum-based combination chemotherapy	—	587/587 (100)	—	—	AlexNet^s^, GoogLeNet^t^, VGGNet^u^, SVM, modern deep convolutional neural networks, and multilayer neural networks	1	—
Yu et al [44], 2016	America	TCGA and CPTAC	Platinum-based combination chemotherapy	35/130 (2)	130/130 (100)	130	LASSO	RF, SVM, NB^v^, and COX	1	—
Sun et al [45], 2016	China	Tongji Hospital and Hubei Cancer Hospital	Platinum or taxane-based chemotherapy	43/100 (43)	100/251 (39.8)	251	Univariate analysis	SVM	2	151
Chen et al [46], 2022	China	TCGA or GEO	Platinum-based combination chemotherapy	161/230 (70)	230/305 (75.4)	305	Univariate analysis	RF and COX	1	75
Li et al [47], 2022	China	TCGA or GEO	Platinum-based combination chemotherapy	287/489 (58.7)	489/797 (61.4)	797	LASSO	LR	1	308
Zhao et al [48], 2021	China	TCGA or GEO	Platinum-based combination chemotherapy	—	146/483 (30.2)	483	—	LR	1	337
Buttarelli et al [49], 2022	Italy	TCGA or GEO	Platinum-based combination chemotherapy	7/14 (50)	14/58 (24)	58	—	RF	1	44
Gonzalez Bosquet et al [50], 2016	America	NCI^w^ and NHGRI^x^	Platinum-based combination chemotherapy	292/450 (64.9)	450/450 (100)	—	Multivariate analysis	RF, Elastic Net^y^, PAM^z^, Diagonal Discriminant Analysis, partial least squares–LR, penalized LR, partial least squares, and partial least squares–RF	1	—
Gong et al [51], 2021	China	Shengjing Hospital of China Medical University	Platinum or paclitaxel-based treatment	77/174 (44)	174/174 (100)	—	—	NB, generalized linear model, LR, Fast Large Margin, deep learning, DT, RF, Gradient Boosting Tree, and SVM	1	—
Sun and Yang [52], 2020	China	TCGA	Platinum-based combination chemotherapy	95/320 (29)	320/320 (100)	—	Univariate and multivariate analysis	LR	—	—
Lei et al [53], 2022	China	The Sun Yat-sen Memorial Hospital	Platinum-based combination chemotherapy	50/62 (80)	62/93 (66)	93	—	Convolutional neural network, principal component analysis, and SVM	1	31

^a^GDSC: Genomics of Drug Sensitivity in Cancer.

^b^TCGA: The Cancer Genome Atlas.

^c^GEO: Gene Expression Omnibus.

^d^Missing data or not applicable.

^e^SVM: support vector machine.

^f^LR: logistic regression.

^g^KNN: k-nearest neighbor.

^h^DT: decision tree.

^i^AdaBoost: Adaptive Boosting.

^j^GBM: Gradient Boosting Machine.

^k^XGBoost: Extreme Gradient Boosting.

^l^RF: random forest.

^m^DNN: deep neural network.

^n^COX: Cox Proportional Hazards Regression and Log-Rank Tests.

^o^ANN: artificial neural network.

^p^SPC: supervised principal component.

^q^LASSO: Least Absolute Shrinkage and Selection Operator.

^r^CPTAC: Clinical Proteomic Tumor Analysis Consortium.

^s^AlexNet: Alexandria Network.

^t^GoogLeNet: Google’s Network.

^u^VGGNet: Visual Geometry Group Network.

^v^NB: naive Bayes.

^w^NCI: National Cancer Institute.

^x^NHGRI: National Human Genome Research Institute.

^y^Elastic Net: Elastic Net Regularization.

^z^PAM: Prediction Analysis of Microarrays.

### Quality Assessment of Included Studies Using PROBAST

**Figure 2:**
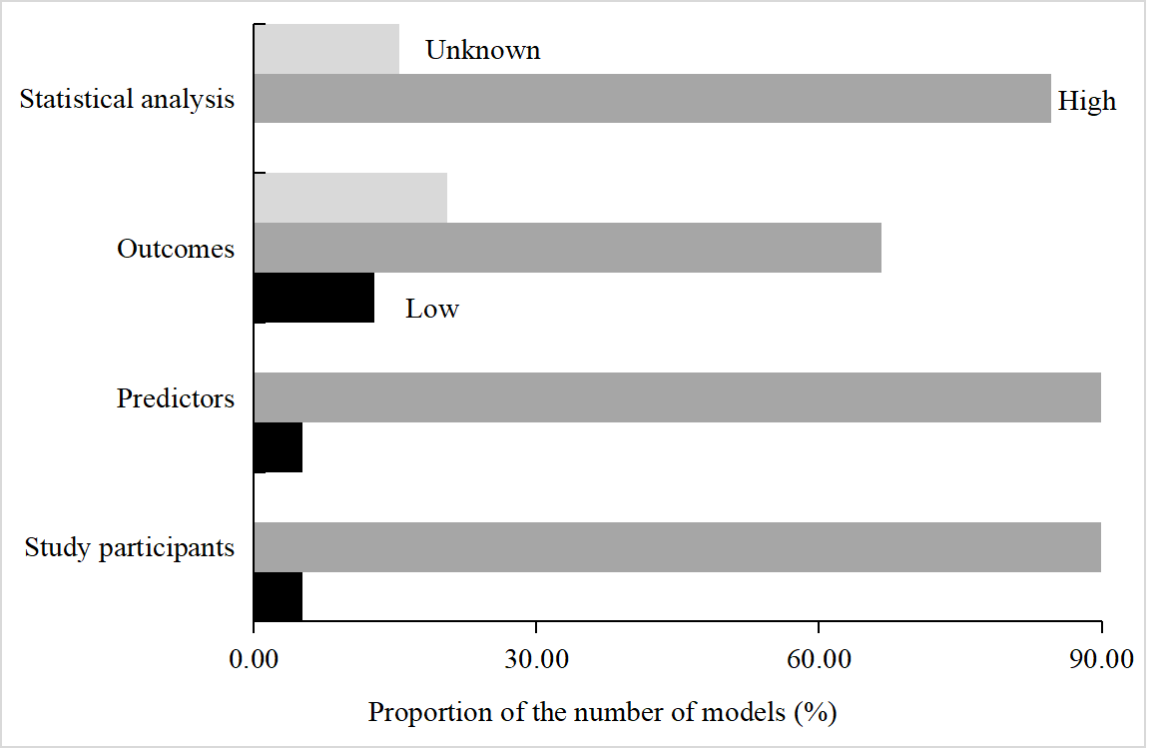
Risk of bias assessment for all eligible studies.

PROBAST was used to evaluate the risk of bias in eligible articles that constituted or externally validated predictive models. Figure 2 summarizes the risk of bias in the 39 predictive models. Overall, 2 models had a low risk of bias in research participants, 2 models had a low risk of bias in predictors, 4 models had a low risk of bias in outcomes, and none had a low risk of bias in statistical analysis (Multimedia Appendix 3). Some models were at a high risk of bias, suggesting that their real predictive performance may be worse than that previously reported. Therefore, we are reasonably concerned that these models may be unreliable when used by others.

### Model Performance Evaluation

**Figure 3:**
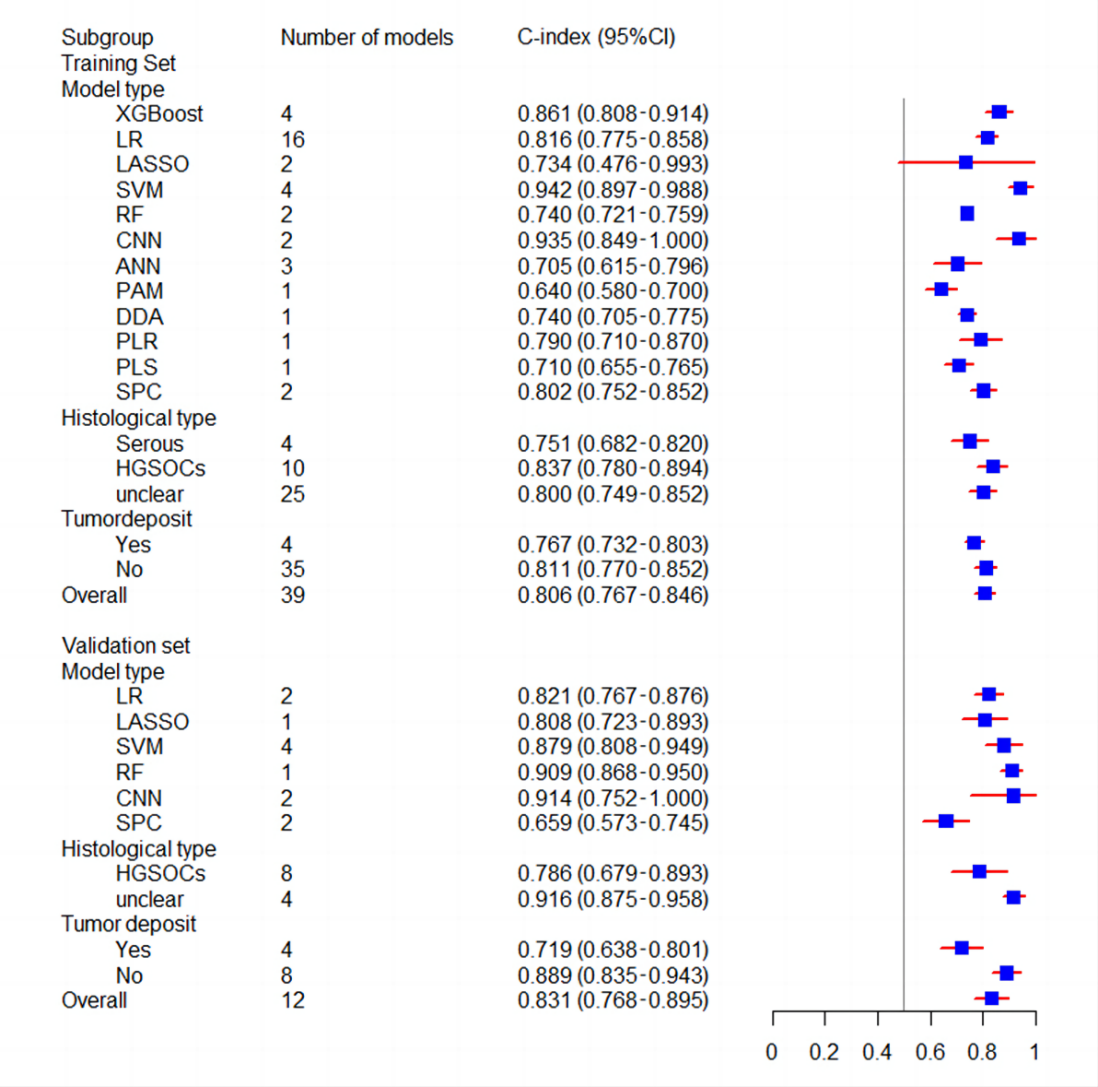
Forest plot of subgroup analysis. ANN: artificial neural network; CNN: convolutional neural network; DDA: Diagonal Discriminant Analysis; HGSOC: high-grade serous ovarian cancer; LASSO: Least Absolute Shrinkage and Selection Operator; LR: logistic regression; PAM: Prediction Analysis of Microarrays; PLR: penalized logistic regression; PLS: partial least squares; RF: random forest; SPC: supervised principal component; SVM: support vector machine; XGBoost: Extreme Gradient Boosting.

**Figure 4:**
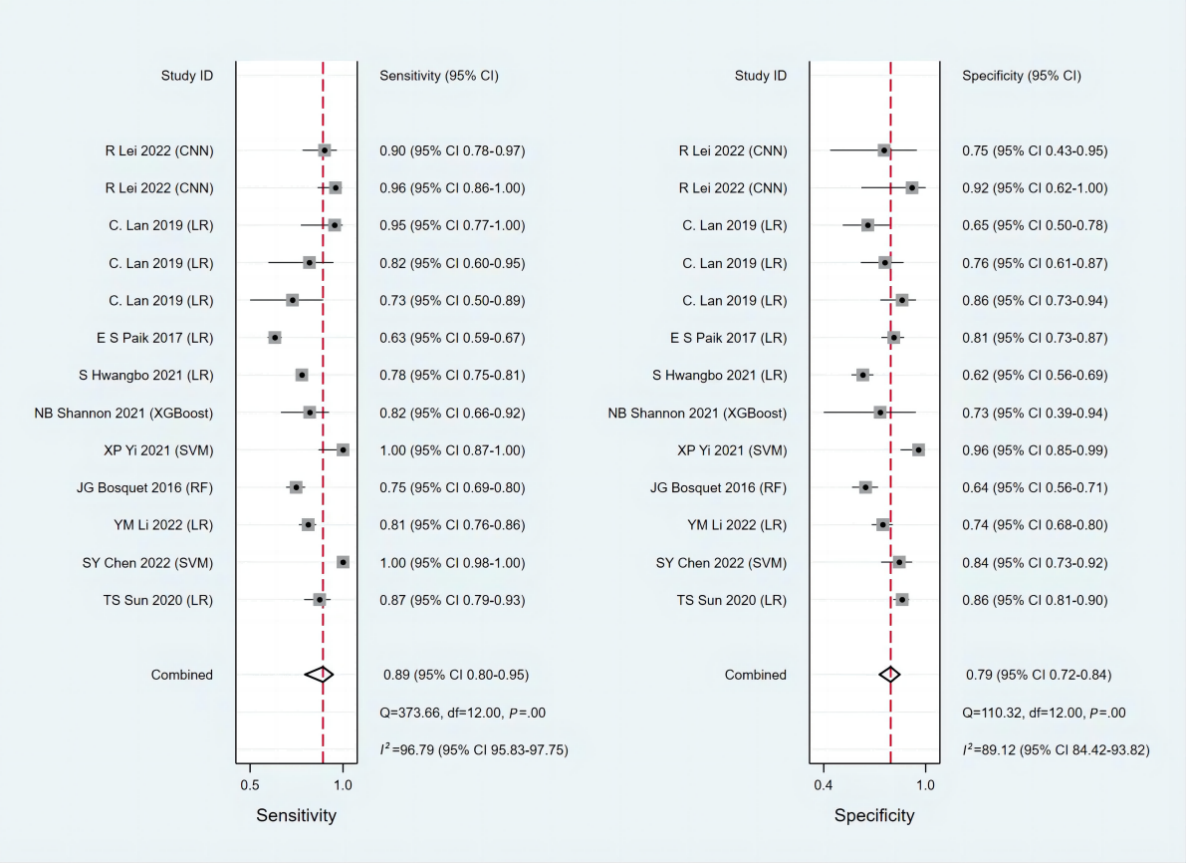
Meta-analysis of sensitivity and specificity—the training set. The repeated authors in the literature are due to the construction of multiple machine learning models. CNN: convolutional neural network; LR: logistic regression; RF: random forest; SVM: support vector machine; XGBoost: Extreme Gradient Boosting.

**Figure 5:**
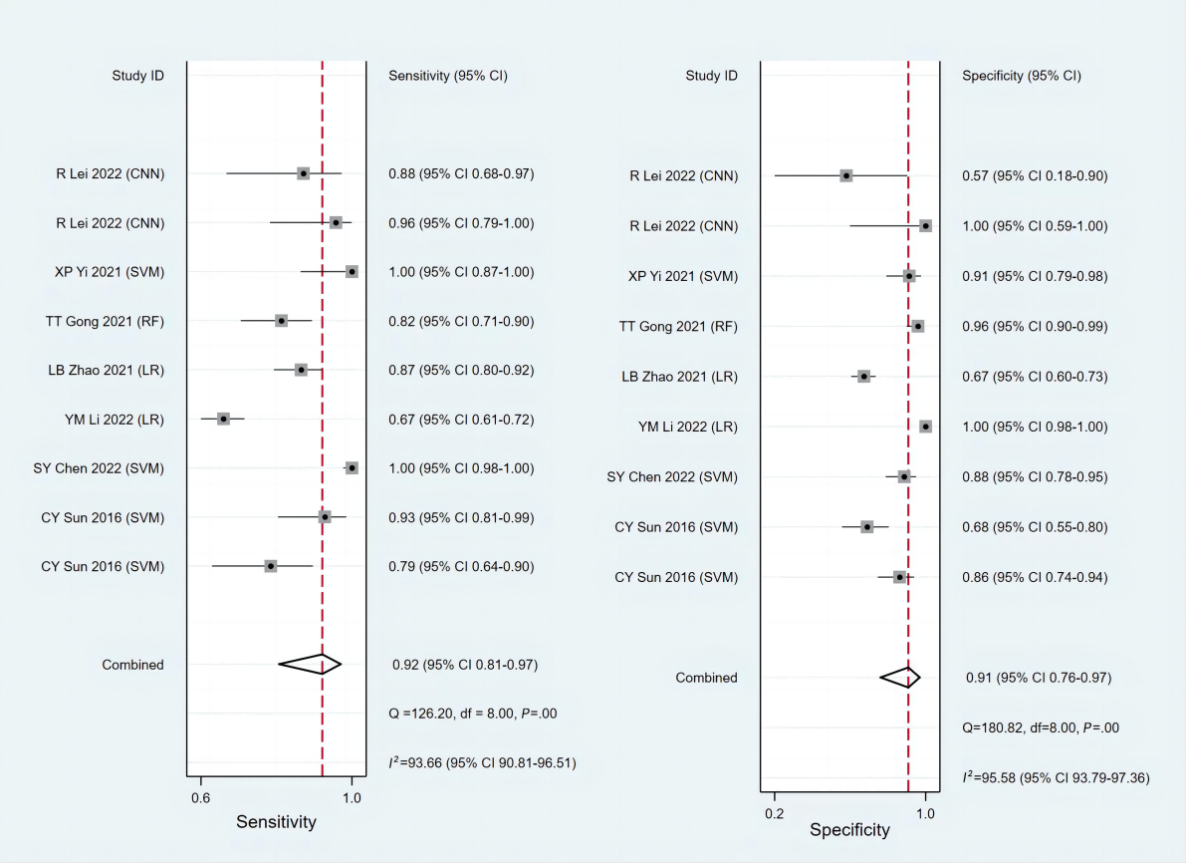
Meta-analysis of sensitivity and specificity—the test set. The repeated authors in the literature are due to the construction of multiple machine learning models. CNN: convolutional neural network; LR: logistic regression; RF: random forest; SVM: support vector machine; XGBoost: Extreme Gradient Boosting.

Discrimination and calibration are the most commonly used indicators for assessing the prediction model performance. Discrimination is usually evaluated by the area under the receiver operating characteristic curve, namely the C-index, which is between 0.5 and 1. A higher C-index indicates better discrimination in the prediction model. In general, a random effects model was used for the meta-analysis of C-index in 39 predictive models (XGBoost, LR, Least Absolute Shrinkage and Selection Operator [LASSO], SVM, random forest, convolutional neural networks, artificial neural networks, Prediction Analysis of Microarrays, Diagonal Discriminant Analysis, Penalized Logistic Regression, partial least squares, and supervised principal components method). The training cohort reported C-index in 39 predictive models, with a pooled value of 0.806 (95% CI 0.767-0.846); the validation cohort reported C-index in 12 predictive models, with a pooled value of 0.831 (95% CI 0.768-0.895). We conducted subgroup analyses according to the machine learning model type, histological type of ovarian cancer, and whether there was residual tumor after surgery. In terms of the subgroup analysis of model types, the C-index for the models in the training set was as follows—XGBoost: 0.861 (95% CI 0.808-0.914), LR: 0.816 (95% CI 0.775-0.858), SVM: 0.942 (95% CI 0.897-0.988), and ANN: 0.705 (95% CI 0.615-0.796); the C-index for the models in the test set were LR: 0.821 (95% CI 0.767-0.876), LASSO: 0.808 (95% CI 0.723-0.893), SVM: 0.879 (95% CI 0.808-0.949), and random forest: 0.909 (95% CI 0.868-0.950). With regard to the subgroup analysis of pathological types, the C-index in the training cohort was serous adenocarcinoma (0.751, 95% CI 0.682-0.820), high-grade serous ovarian cancers (0.837, 95% CI 0.780-0.894), and unclear (0.800, 95% CI 0.749-0.852); the C-index in the test set was high-grade serous ovarian cancers (0.786, 95% CI 0.679-0.893) and unclear (0.916, 95% CI 0.875-0.958). Meanwhile, in the subgroup analysis of residual tumor, the C-index for residual tumor in the training cohort was 0.767 (95% CI 0.732-0.803) and the C-index for nonresidual tumor was 0.811 (95% CI 0.770-0.852). In the test set, the C-index for residual tumor was 0.719 (95% CI 0.638-0.801) and the C-index for nonresidual tumor was 0.889 (95% CI 0.835-0.943). The forest plot for the subgroup analysis is shown in Figure 3. Table 2 presents the meta-analysis results of the C-index. High heterogeneity was identified among these studies (*I*^2^=97.3%; *P*≤.001), probably because of the varied machine learning methods and variables used in these studies. Furthermore, a meta-analysis of the sensitivity and specificity of the 22 predictive models was performed. The pooled sensitivity was 0.890 (95% CI 0.800-0.950) and the pooled specificity was 0.790 (95% CI 0.720-0.840) in the training set (Figure 4) [35-37,39,42,46,47,50,52,53]. In the test set, the pooled sensitivity was 0.920 (95% CI 0.810-0.970) and the pooled specificity was 0.910 (95% CI 0.760-0.970; Figure 5) [42,45-48,51,53].

**Table 2:** Meta-analysis results of predicting platinum chemotherapy responses in ovarian cancer.

		Training set		Test set	
		Number (n=39), n (%)	C-index (95% CI)	Number (n=12), n (%)	C-index (95% CI)
**Model**					
	XGBoost^a^	4 (10)	0.861 (0.808-0.914)	—^b^	—
	LR^c^	16 (41)	0.816 (0.775-0.858)	2 (17)	0.821 (0.767-0.876)
	LASSO^d^	2 (5)	0.734 (0.476-0.993)	1 (8)	0.808 (0.723-0.893)
	SVM^e^	4 (10)	0.942 (0.897-0.988)	4 (33)	0.879 (0.808-0.949)
	RF^f^	2 (5)	0.740 (0.721-0.759)	1 (8)	0.909 (0.868-0.950)
	CNN^g^	2 (5)	0.935 (0.849-1.000)	2 (17)	0.914 (0.752-1.000)
	ANN^h^	3 (8)	0.705 (0.615-0.796)	—	—
	PAM^i^	1 (3)	0.640 (0.580-0.700)	—	—
	DDA^j^	1 (3)	0.740 (0.705-0.775)	—	—
	PLR^k^	1 (3)	0.790 (0.710-0.870)	—	—
	PLS^l^	1 (3)	0.710 (0.655-0.765)	—	—
	SPC^m^	2 (5)	0.802 (0.752-0.852)	2 (17)	0.659 (0.573-0.745)
**Histological type**					
	Serous	4 (10)	0.751 (0.682-0.820)	—	—
	HGSOC^n^	10 (26)	0.837 (0.780-0.894)	8 (67)	0.786 (0.679-0.893)
	Unclear	25 (64)	0.800 (0.749-0.852)	4 (33)	0.916 (0.875-0.958)
**Residual tumor**					
	Yes	4 (10)	0.767 (0.732-0.803)	4 (33)	0.719 (0.638-0.801)
	No	35 (90)	0.811 (0.770-0.852)	8 (67)	0.889 (0.835-0.943)
Overall		39 (100)	0.806 (0.767-0.846)	12 (100)	0.831 (0.768-0.895)

^a^XGBoost: Extreme Gradient Boosting.

^b^Missing data.

^c^LR: logistic regression.

^d^LASSO: Least Absolute Shrinkage and Selection Operator.

^e^SVM: support vector machine.

^f^RF: random forest.

^g^CNN: convolutional neural network.

^h^ANN: artificial neural network.

^i^PAM: Prediction Analysis of Microarrays.

^j^DDA: Diagonal Discriminant Analysis.

^k^PLR: penalized logistic regression.

^l^PLS: partial least squares.

^m^SPC: supervised principal component.

^n^HGSOC: high-grade serous ovarian cancer.

## Discussion

### Principal Findings

This study conducted a meta-analysis of machine learning models for predicting responses to platinum chemotherapy in patients with ovarian cancer. It delves into the performance, reliability, and influencing factors of models. To our knowledge, this is the first systematic review and meta-analysis on the application of machine learning in predicting responses to platinum-based chemotherapy in patients with ovarian cancer. The search initially yielded 1749 studies, and after applying inclusion criteria, 19 studies (accounting for 1.09% of the total) were ultimately included. This research encompasses 12 machine learning models, such as XGBoost, LR, LASSO, and SVM, built based on various hospital or genomics data sources. The analysis results indicated that these models performed effectively in distinguishing patients’ responses to platinum chemotherapy, achieving C-indices of 0.806 and 0.831 in the training and validation sets, respectively. The model demonstrated high overall sensitivity and specificity, underscoring its accuracy and reliability in predicting platinum drug response in ovarian cancer. Subgroup analysis revealed the influence of model type, pathology type, and residual tumor on the prediction performance. SVM stood out on both the training and validation sets because it outperformed other machine learning methods in terms of accuracy and relative error rate measures [54] and exhibited the ability to identify subtle patterns in complex data sets [55]. LR is the most commonly used modeling variable because it can handle not only binary results but also accommodate continuous or categorical predictor variables. This comprehensive approach considers the impact of multiple factors on the results, effectively controls potential confounding factors, and reduces bias [56]. As a result, LR is widely used in machine learning modeling within various fields. The analysis of residual tumor revealed that the model exhibited different performance in predicting patients with or without residual tumor. Compared with nonresidual tumor, the predictive performance of machine learning for residual tumor was more significant, suggesting that residual tumor may be a crucial factor influencing ovarian cancer patients’ response to platinum therapy.

Most published meta-analyses on the application of machine learning in ovarian cancer focus on the diagnosis and prediction of ovarian cancer; however, there are some differences in specific research methods, evaluation tools, and presentation of results. Huang et al [57] reviewed the application of computed tomography and magnetic resonance imaging radiomics in ovarian cancer, achieving promising results in differential diagnosis and prognosis prediction. Other studies [58,59] have summarized artificial intelligence methods for gynecological malignant tumors, emphasizing that variable selection, machine learning methods, and end point selection can all influence model performance. Xu et al [60] systematically reviewed studies that applied artificial intelligence to diagnose ovarian cancer based on medical images and highlighted the good performance of artificial intelligence algorithms in ovarian cancer diagnosis. Koch et al [61] evaluated the accuracy of computer-aided diagnosis, encompassing computer-aided diagnosis for ultrasound, computed tomography, and magnetic resonance imaging, to predict the likelihood of malignancy in ovarian tumors. Given that it is challenging to predict the response of patients with ovarian cancer to platinum therapy before the completion of chemotherapy, accurate prediction of this response is crucial for devising effective treatment plans. This review focuses on the performance of machine learning in predicting responses to platinum-based chemotherapy in patients with ovarian cancer. This not only provides valuable information for clinical prediction but also addresses a long-standing challenge in the development of noninvasive methods for predicting chemotherapy response in patients with ovarian cancer. Feature selection emerges as a critical aspect influencing model performance in this context. Previous studies [41,62] have reported that next-generation sequencing technology can be used to explore correlations between intrinsic genomic features and the response to platinum-based chemotherapy. Radiomics is another approach. A recent study demonstrated that a predictive model based on the combination of radiomics with single nucleotide polymorphisms of Human Sulfatase 1 could predict platinum resistance in ovarian cancer treatment [42]. Previous research [43] has shown that combining whole-slide histopathology scanners and high-throughput omics analysis with cutting-edge machine learning algorithms can help reveal correlations between microscopic tumor cell morphology and molecular pathways. Machine learning models have shown great promise in linking histopathological patterns to patient diagnosis and prognosis. Another study [44] used tumor proteomic features to predict the clinical response to platinum-based chemotherapy in patients with ovarian cancer. The findings revealed a close association between tissue expression levels of 24 proteins and the response to platinum-based chemotherapy. The variables selected in the 19 included articles spanned from molecular-level factors to clinical characteristics, medical imaging, and the microbiome, reflecting the prevailing trend of considering multiple levels and aspects in cancer research. This comprehensive approach facilitates a more in-depth understanding of cancer pathogenesis and predictive factors.

### Strengths and Limitations

#### Strengths

The most noteworthy aspect of our analysis is that it provides a comprehensive map of research on prognostic prediction models for patients with ovarian cancer. We gathered all available predictive models for potential clinical outcomes of platinum chemotherapy responses in patients with ovarian cancer. The characteristics of these models were elucidated in detail. Furthermore, this study critically evaluated the predictive models for platinum chemotherapy response in patients with ovarian cancer using the PROBAST tool. Moreover, a meta-analysis of the C-index using multiple externally verified predictive models was performed. There is currently no meta-analysis that summarizes research on machine learning prediction models for platinum chemotherapy response in ovarian cancer. Hence, this study aimed to explore its performance in prediction. It is critical to systematically review published studies on machine learning and provide guidance for future research. This helps to establish personalized treatment protocols and estimate prognosis by elucidating intrinsic tumor features such as platinum sensitivity in the initial therapy.

#### Limitations

However, several limitations of the current investigation must be considered. First, the meta-analysis of the C-index had a high degree of heterogeneity, probably because of the various machine learning methods, predictors, and parameters used in model construction, as well as differences in clinical settings, patient characteristics, and research time. At the same time, we should note that the risk of bias assessment of the predictive model is a rigorous tool for the construction of original models; for most of the original studies, the results assessed by this tool have a high risk of bias. In addition, our meta-analysis had several methodological problems in model development, which were reflected in the risk of bias. The PROBAST assessment suggested that some studies had a high or unclear risk of bias in 4 domains: participants, predictors, outcomes, and statistical analysis. Furthermore, the predictive value of machine learning for different diseases may vary. The essence of machine learning is efficient predictors. When the same machine learning model includes more efficient predictors, its predictive value will be significantly improved. This may result in heterogeneity between models. For constructing machine learning, especially for rare events, some studies face challenges in acquiring large data sets, making it difficult to establish an independent validation set. However, the importance of the training process cannot be overlooked as cross-validation may be used during training, although it cannot fully replace an independent validation set. When conducting meta-analysis, it is essential to consider whether the model is overfitting, necessitating attention to the results of the training set. Consequently, our meta-analysis includes studies without independent validation cohorts for a comprehensive evaluation. The most important aspect is the lack of original research with large multicenter samples in the modeling process. Therefore, more high-quality, multicenter, large-scale studies are required. Despite some limitations in this study, we have compiled a comprehensive summary of the current models to provide a reference for the development of more broadly applicable clinical tools in the future. Looking at it this way, it is necessary to conduct a meta-analysis. Although there are frequent disagreements about the predictive value of different studies, this is partially dependent on the selection of the predictive model, which is the most influential factor affecting predictive performance.

### Conclusions

Machine learning has excellent predictive performance in predicting response to platinum chemotherapy in patients with ovarian cancer. At the same time, we found that SVM has the best prediction performance among the existing prediction models. Machine learning can be used as a prediction tool for platinum response in ovarian cancer. On the basis of this research, a large-scale, multicenter, and multiethnic prediction tool can be developed in the future for predicting platinum-based chemotherapy response in patients with ovarian cancer to advance precision chemotherapy for ovarian cancer management.

## Appendix

Multimedia Appendix 1 PRISMA (Preferred Reporting Items for Systematic Reviews and Meta-Analyses) 2020 checklist.

Multimedia Appendix 2 The specific retrieval strategy of this paper.

Multimedia Appendix 3 Results of bias risk assessment through Prediction Model Risk of Bias Assessment in 19 included articles.

## Abbreviations

C-index: concordance index
EOC: epithelial ovarian cancer
LASSO: Least Absolute Shrinkage and Selection Operator
LR: logistic regression
PRISMA: Preferred Reporting Items for Systematic Reviews and Meta-Analyses
PROBAST: Prediction Model Risk of Bias Assessment
SVM: support vector machine
XGBoost: Extreme Gradient Boosting

## Data Availability

The original contributions presented in this study are included in the paper or multimedia appendices, and further inquiries can be directed to the corresponding author.

## References

[1] Zachou G, El-Khouly F, Dilley J (2023). Evaluation of follow-up strategies for women with epithelial ovarian cancer following completion of primary treatment. Cochrane Database Syst Rev.

[2] Coburn SB, Bray F, Sherman ME, Trabert B (2017). International patterns and trends in ovarian cancer incidence, overall and by histologic subtype. Int J Cancer.

[3] Retamales-Ortega R, Oróstica Lorena, Vera C, Cuevas P, Hernández Andrea, Hurtado I, Vega M, Romero C (2017). Role of Nerve Growth Factor (NGF) and miRNAs in Epithelial Ovarian Cancer. Int J Mol Sci.

[4] Zhu JW, Charkhchi P, Akbari MR (2022). Potential clinical utility of liquid biopsies in ovarian cancer. Mol Cancer.

[5] Torre LA, Trabert B, DeSantis CE, Miller KD, Samimi G, Runowicz CD, Gaudet MM, Jemal A, Siegel RL (2018). Ovarian cancer statistics, 2018. CA Cancer J Clin.

[6] Kania KD, Haręża D, Wilczyński JR, Wilczyński M, Jarych D, Malinowski A, Paradowska E (2022). The toll-like receptor 4 polymorphism Asp299Gly is associated with an increased risk of ovarian cancer. Cells.

[7] Lheureux S, Gourley C, Vergote I, Oza AM (2019). Epithelial ovarian cancer. Lancet.

[8] Cabasag CJ, Fagan PJ, Ferlay J, Vignat J, Laversanne M, Liu L, van der Aa MA, Bray F, Soerjomataram I (2022). Ovarian cancer today and tomorrow: a global assessment by world region and Human Development Index using GLOBOCAN 2020. Int J Cancer.

[9] Xu Y, Liu H, Chen J, Zhou X NCCN clinical practice guidelines in oncology: ovarian cancer continue including fallopian tube cancer and primary peritoneal cancer(version 2.2023). National Comprehensive Cancer Network.

[10] Noriega-Rivera R, Rivera-Serrano M, Rabelo-Fernandez RJ, Pérez-Santiago J, Valiyeva F, Vivas-Mejía PE (2022). Upregulation of the long noncoding RNA CASC10 promotes cisplatin resistance in high-grade serous ovarian cancer. Int J Mol Sci.

[11] Bogani G, Rossetti D, Ditto A, Martinelli F, Chiappa V, Mosca L, Leone Roberti Maggiore U, Ferla S, Lorusso D, Raspagliesi F (2018). Artificial intelligence weights the importance of factors predicting complete cytoreduction at secondary cytoreductive surgery for recurrent ovarian cancer. J Gynecol Oncol.

[12] Kurtz JE, Pujade-Lauraine E, Oaknin A, Belin L, Leitner K, Cibula D, Denys H, Rosengarten O, Rodrigues M, de Gregorio N, Martinez García J, Petru E, Kocián R, Vergote I, Pautier P, Schmalfeldt B, Gaba L, Polterauer S, Mouret Reynier M, Sehouli J, Churruca C, Selle F, Joly F, D'Hondt V, Bultot-Boissier É, Lebreton C, Lotz JP, Largillier R, Heudel P, Heitz F, ATALANTE/ENGOT-ov29 Investigators (2023). Atezolizumab combined with bevacizumab and platinum-based therapy for platinum-sensitive ovarian cancer: placebo-controlled randomized phase III ATALANTE/ENGOT-ov29 trial. J Clin Oncol.

[13] Wilson MK, Pujade-Lauraine E, Aoki D, Mirza MR, Lorusso D, Oza AM, du Bois A, Vergote I, Reuss A, Bacon M, Friedlander M, Gallardo-Rincon D, Joly F, Chang SJ, Ferrero AM, Edmondson RJ, Wimberger P, Maenpaa J, Gaffney D, Zang R, Okamoto A, Stuart G, Ochiai K, participants of the Fifth Ovarian Cancer Consensus Conference (2017). Fifth Ovarian Cancer Consensus Conference of the Gynecologic Cancer InterGroup: recurrent disease. Ann Oncol.

[14] Cancer Genome Atlas Research Network (2011). Integrated genomic analyses of ovarian carcinoma. Nature.

[15] da Costa AA, Baiocchi G (2021). Genomic profiling of platinum-resistant ovarian cancer: the road into druggable targets. Semin Cancer Biol.

[16] Chatterjee N, Bivona TG (2019). Polytherapy and targeted cancer drug resistance. Trends Cancer.

[17] Borley J, Brown R (2015). Epigenetic mechanisms and therapeutic targets of chemotherapy resistance in epithelial ovarian cancer. Ann Med.

[18] Natanzon Y, Goode EL, Cunningham JM (2018). Epigenetics in ovarian cancer. Semin Cancer Biol.

[19] Kleih M, Böpple Kathrin, Dong M, Gaißler Andrea, Heine S, Olayioye MA, Aulitzky WE, Essmann F (2019). Direct impact of cisplatin on mitochondria induces ROS production that dictates cell fate of ovarian cancer cells. Cell Death Dis.

[20] Han Y, Kim B, Cho U, Park IS, Kim SI, Dhanasekaran DN, Tsang BK, Song YS (2019). Mitochondrial fission causes cisplatin resistance under hypoxic conditions via ROS in ovarian cancer cells. Oncogene.

[21] Lheureux S, Braunstein M, Oza AM (2019). Epithelial ovarian cancer: evolution of management in the era of precision medicine. CA Cancer J Clin.

[22] Funingana IG, Reinius MA, Petrillo A, Ang JE, Brenton JD (2021). Can integrative biomarker approaches improve prediction of platinum and PARP inhibitor response in ovarian cancer?. Semin Cancer Biol.

[23] Yearley AG, Goedmakers CM, Panahi A, Doucette J, Rana A, Ranganathan K, Smith TR (2023). FDA-approved machine learning algorithms in neuroradiology: a systematic review of the current evidence for approval. Artif Intell Med.

[24] Kumari J, Kumar E, Kumar D (2023). A structured analysis to study the role of machine learning and deep learning in the healthcare sector with big data analytics. Arch Comput Methods Eng.

[25] Feldner-Busztin D, Firbas Nisantzis P, Edmunds SJ, Boza G, Racimo F, Gopalakrishnan S, Limborg MT, Lahti L, de Polavieja GG (2023). Dealing with dimensionality: the application of machine learning to multi-omics data. Bioinformatics.

[26] Monaco A, Pantaleo E, Amoroso N, Lacalamita A, Lo Giudice C, Fonzino A, Fosso B, Picardi E, Tangaro S, Pesole G, Bellotti R (2021). A primer on machine learning techniques for genomic applications. Comput Struct Biotechnol J.

[27] Li Y, Wu X, Yang P, Jiang G, Luo Y (2022). Machine learning for lung cancer diagnosis, treatment, and prognosis. Genomics Proteomics Bioinformatics.

[28] Li G, Li L, Li Y, Qian Z, Wu F, He Y, Jiang H, Li R, Wang D, Zhai Y, Wang Z, Jiang T, Zhang J, Zhang W (2022). An MRI radiomics approach to predict survival and tumour-infiltrating macrophages in gliomas. Brain.

[29] Li X, Dowling EK, Yan G, Dereli Z, Bozorgui B, Imanirad P, Elnaggar JH, Luna A, Menter DG, Pilié PG, Yap TA, Kopetz S, Sander C, Korkut A (2022). Precision combination therapies based on recurrent oncogenic coalterations. Cancer Discov.

[30] Issa NT, Stathias V, Schürer S, Dakshanamurthy S (2021). Machine and deep learning approaches for cancer drug repurposing. Semin Cancer Biol.

[31] Kong J, Lee H, Kim D, Han SK, Ha D, Shin K, Kim S (2020). Network-based machine learning in colorectal and bladder organoid models predicts anti-cancer drug efficacy in patients. Nat Commun.

[32] Moons KG, Wolff RF, Riley RD, Whiting PF, Westwood M, Collins GS, Reitsma JB, Kleijnen J, Mallett S (2019). PROBAST: a tool to assess risk of bias and applicability of prediction model studies: explanation and elaboration. Ann Intern Med.

[33] Nagendran M, Chen Y, Lovejoy CA, Gordon AC, Komorowski M, Harvey H, Topol EJ, Ioannidis JP, Collins GS, Maruthappu M (2020). Artificial intelligence versus clinicians: systematic review of design, reporting standards, and claims of deep learning studies. BMJ.

[34] Debray TP, Damen JA, Riley RD, Snell K, Reitsma JB, Hooft L, Collins GS, Moons KG (2019). A framework for meta-analysis of prediction model studies with binary and time-to-event outcomes. Stat Methods Med Res.

[35] Shannon NB, Tan LL, Tan QX, Tan JW, Hendrikson J, Ng WH, Ng G, Liu Y, Ong XS, Nadarajah R, Wong JS, Tan GH, Soo KC, Teo MC, Chia CS, Ong CJ (2021). A machine learning approach to identify predictive molecular markers for cisplatin chemosensitivity following surgical resection in ovarian cancer. Sci Rep.

[36] Lan C, Li J, Huang X, Heindl A, Wang Y, Yan S, Yuan Y (2019). Stromal cell ratio based on automated image analysis as a predictor for platinum-resistant recurrent ovarian cancer. BMC Cancer.

[37] Hwangbo S, Kim SI, Kim JH, Eoh KJ, Lee C, Kim YT, Suh DS, Park T, Song YS (2021). Development of machine learning models to predict platinum sensitivity of high-grade serous ovarian carcinoma. Cancers (Basel).

[38] Zhao H, Sun Q, Li L, Zhou J, Zhang C, Hu T, Zhou X, Zhang L, Wang B, Li B, Zhu T, Li H (2019). High expression levels of AGGF1 and MFAP4 predict primary platinum-based chemoresistance and are associated with adverse prognosis in patients with serous ovarian cancer. J Cancer.

[39] Paik ES, Sohn I, Baek S, Shim M, Choi HJ, Kim T, Choi CH, Lee J, Kim B, Lee Y, Bae D (2017). Nomograms predicting platinum sensitivity, progression-free survival, and overall survival using pretreatment complete blood cell counts in epithelial ovarian cancer. Cancer Res Treat.

[40] Han Y, Huang H, Xiao Z, Zhang W, Cao Y, Qu L, Shou C (2012). Integrated analysis of gene expression profiles associated with response of platinum/paclitaxel-based treatment in epithelial ovarian cancer. PLoS One.

[41] Zheng H, Shu T, Zhu S, Zhang C, Gao M, Zhang N, Wang H, Yuan J, Tai Z, Xia X, Yi Y, Li J, Guan Y, Xiang Y, Gao Y (2021). Construction and validation of a platinum sensitivity predictive model with multiple genomic variations for epithelial ovarian cancer. Front Oncol.

[42] Yi X, Liu Y, Zhou B, Xiang W, Deng A, Fu Y, Zhao Y, Ouyang Q, Liu Y, Sun Z, Zhang K, Li X, Zeng F, Zhou H, Chen BT (2021). Incorporating SULF1 polymorphisms in a pretreatment CT-based radiomic model for predicting platinum resistance in ovarian cancer treatment. Biomed Pharmacother.

[43] Yu K, Hu V, Wang F, Matulonis UA, Mutter GL, Golden JA, Kohane IS (2020). Deciphering serous ovarian carcinoma histopathology and platinum response by convolutional neural networks. BMC Med.

[44] Yu KH, Levine DA, Zhang H, Chan DW, Zhang Z, Snyder M (2016). Predicting ovarian cancer patients' clinical response to platinum-based chemotherapy by their tumor proteomic signatures. J Proteome Res.

[45] Sun CY, Su TF, Li N, Zhou B, Guo ES, Yang ZY, Liao J, Ding D, Xu Q, Lu H, Meng L, Wang SX, Zhou JF, Xing H, Weng DH, Ma D, Chen G (2016). A chemotherapy response classifier based on support vector machines for high-grade serous ovarian carcinoma. Oncotarget.

[46] Chen S, Wu Y, Wang S, Wu J, Wu X, Zheng Z (2022). A risk model of gene signatures for predicting platinum response and survival in ovarian cancer. J Ovarian Res.

[47] Li Y, Nie Y, Guo H, Guo H, Ha C, Li Y (2022). Establish of an initial platinum-resistance predictor in high-grade serous ovarian cancer patients regardless of homologous recombination deficiency status. Front Oncol.

[48] Zhao L, Ma S, Wang L, Wang Y, Feng X, Liang D, Han L, Li M, Li Q (2021). A polygenic methylation prediction model associated with response to chemotherapy in epithelial ovarian cancer. Mol Ther Oncolytics.

[49] Buttarelli M, Ciucci A, Palluzzi F, Raspaglio G, Marchetti C, Perrone E, Minucci A, Giacò L, Fagotti A, Scambia G, Gallo D (2022). Identification of a novel gene signature predicting response to first-line chemotherapy in BRCA wild-type high-grade serous ovarian cancer patients. J Exp Clin Cancer Res.

[50] Gonzalez Bosquet J, Newtson AM, Chung RK, Thiel KW, Ginader T, Goodheart MJ, Leslie KK, Smith BJ (2016). Prediction of chemo-response in serous ovarian cancer. Mol Cancer.

[51] Gong TT, He XH, Gao S, Wu QJ (2021). Application of machine learning in prediction of chemotherapy resistant of ovarian cancer based on gut microbiota. J Cancer.

[52] Sun T, Yang Q (2020). Chemoresistance-associated alternative splicing signatures in serous ovarian cancer. Oncol Lett.

[53] Lei R, Yu Y, Li Q, Yao Q, Wang J, Gao M, Wu Z, Ren W, Tan Y, Zhang B, Chen L, Lin Z, Yao H (2022). Deep learning magnetic resonance imaging predicts platinum sensitivity in patients with epithelial ovarian cancer. Front Oncol.

[54] Nedaie A, Najafi AA (2018). Support vector machine with Dirichlet feature mapping. Neural Netw.

[55] Huang S, Cai N, Pacheco PP, Narrandes S, Wang Y, Xu W (2018). Applications of support vector machine (SVM) learning in cancer genomics. Cancer Genomics Proteomics.

[56] LaValley MP (2008). Logistic regression. Circulation.

[57] Huang ML, Ren J, Jin ZY, Liu XY, He YL, Li Y, Xue HD (2023). A systematic review and meta-analysis of CT and MRI radiomics in ovarian cancer: methodological issues and clinical utility. Insights Imaging.

[58] Shrestha P, Poudyal B, Yadollahi S, E Wright D, V Gregory A, D Warner J, Korfiatis P, C Green I, L Rassier S, Mariani A, Kim B, Laughlin-Tommaso SK, L Kline T (2022). A systematic review on the use of artificial intelligence in gynecologic imaging - background, state of the art, and future directions. Gynecol Oncol.

[59] Sheehy J, Rutledge H, Acharya UR, Loh HW, Gururajan R, Tao X, Zhou X, Li Y, Gurney T, Kondalsamy-Chennakesavan S (2023). Gynecological cancer prognosis using machine learning techniques: a systematic review of the last three decades (1990-2022). Artif Intell Med.

[60] Xu HL, Gong TT, Liu FH, Chen HY, Xiao Q, Hou Y, Huang Y, Sun HZ, Shi Y, Gao S, Lou Y, Chang Q, Zhao YH, Gao QL, Wu QJ (2022). Artificial intelligence performance in image-based ovarian cancer identification: a systematic review and meta-analysis. EClinicalMedicine.

[61] Koch AH, Jeelof LS, Muntinga CL, Gootzen TA, van de Kruis NM, Nederend J, Boers T, van der Sommen F, Piek JM (2023). Analysis of computer-aided diagnostics in the preoperative diagnosis of ovarian cancer: a systematic review. Insights Imaging.

[62] Kuhlmann JD, Chebouti I, Kimmig R, Buderath P, Reuter M, Puppel SH, Wimberger P, Kasimir-Bauer S (2019). Extracellular vesicle-associated miRNAs in ovarian cancer - design of an integrated NGS-based workflow for the identification of blood-based biomarkers for platinum-resistance. Clin Chem Lab Med.

