# Supplementary material for: Predictive Value of Machine Learning for Platinum Chemotherapy Responses in Ovarian Cancer: Systematic Review and Meta-Analysis
Source: J Med Internet Res. 2024 Jan 22;26:e48527. doi: 10.2196/48527 (PMC10845031; doi:10.2196/48527)
Supplement: Multimedia Appendix 2 [file jmir_v26i1e48527_app2.docx]

**Table S1 Literature search strategy**

**1.Pubmed**

| Search number | Query | Results |
| --- | --- | --- |
| #1 | ("platinum based chemotherapy"[Title/Abstract] OR "platinum chemotherapy"[Title/Abstract] OR "platinum therapy"[Title/Abstract] OR "platinum reactions"[Title/Abstract] OR "Platinum-Sensitive"[Title/Abstract] OR "Platinum-resistant"[Title/Abstract] OR "platinum hypersensitivity reactions"[Title/Abstract]) | 8,777 |
| #2 | ("Ovarian Neoplasms"[MeSH Terms] OR ("neoplasm ovarian"[Title/Abstract] OR "ovarian neoplasm"[Title/Abstract] OR "ovary neoplasms"[Title/Abstract] OR "neoplasm ovary"[Title/Abstract] OR "neoplasms ovary"[Title/Abstract] OR "ovary neoplasm"[Title/Abstract] OR "neoplasms ovarian"[Title/Abstract] OR "ovary cancer"[Title/Abstract] OR "cancer ovary"[Title/Abstract] OR "cancers ovary"[Title/Abstract] OR "ovary cancers"[Title/Abstract] OR "ovarian cancer"[Title/Abstract] OR "cancer ovarian"[Title/Abstract] OR "cancers ovarian"[Title/Abstract] OR "ovarian cancers"[Title/Abstract] OR "cancer of ovary"[Title/Abstract] OR "cancer of the ovary"[Title/Abstract])) | 110,308 |
| #3 | ("Machine Learning"[MeSH Terms] OR ("deep learning"[Title/Abstract] OR "artificial intelligence"[Title/Abstract] OR "prediction model"[Title/Abstract] OR "transfer learning"[Title/Abstract] OR "random forest"[Title/Abstract] OR "artificial neural network"[Title/Abstract] OR "ANN"[Title/Abstract] OR "support vector machine"[Title/Abstract] OR "SVM"[Title/Abstract] OR "Nomogram"[Title/Abstract] OR "XGboost"[Title/Abstract] OR "Logistic"[Title/Abstract] OR "decision tree"[Title/Abstract] OR "c-index"[Title/Abstract] OR "ROC"[Title/Abstract] OR "AUC"[Title/Abstract] OR "external validation"[Title/Abstract])) | 630,100 |
| #4 | #1 AND #2 AND #3 | 308 |

**2.Cochrane**

| Search number | Query | Results |
| --- | --- | --- |
| #1 | #1 MeSH descriptor: [Ovarian Neoplasms] explode all trees | 2140 |
| #2 | (Neoplasm, Ovarian):ti,ab,kw OR (Ovarian Neoplasm):ti,ab,kw OR (Ovary Neoplasms):ti,ab,kw OR (Neoplasm, Ovary):ti,ab,kw OR (Neoplasms, Ovary):ti,ab,kw | 1555 |
| #3 | (Ovary Neoplasm):ti,ab,kw OR (Neoplasms, Ovarian):ti,ab,kw OR (Ovary Cancer):ti,ab,kw OR (Cancer, Ovary):ti,ab,kw OR (Cancers, Ovary):ti,ab,kw | 5730 |
| #4 | (Ovary Cancers):ti,ab,kw OR (Ovarian Cancer):ti,ab,kw OR (Cancer, Ovarian):ti,ab,kw OR (Cancers, Ovarian):ti,ab,kw OR (Ovarian Cancers):ti,ab,kw | 7586 |
| #5 | (Cancer of Ovary):ti,ab,kw OR (Cancer of the Ovary):ti,ab,kw | 3257 |
| #6 | #1 OR #2 OR #3 OR #4 OR #5 | 8455 |
| #7 | MeSH descriptor: [Machine Learning] explode all trees | 200 |
| #8 | (Deep learning):ti,ab,kw OR (artificial intelligence):ti,ab,kw OR (Prediction model):ti,ab,kw OR (Transfer Learning):ti,ab,kw OR (random forest):ti,ab,kw | 8127 |
| #9 | (artificial neural network):ti,ab,kw OR (ANN):ti,ab,kw OR (Support vector machine):ti,ab,kw OR (SVM):ti,ab,kw OR (Nomogram):ti,ab,kw | 3599 |
| #10 | (XGboost):ti,ab,kw OR (Logistic):ti,ab,kw OR (Decision tree):ti,ab,kw OR (c-index):ti,ab,kw OR (ROC):ti,ab,kw | 31843 |
| #11 | (AUC):ti,ab,kw OR (External validation):ti,ab,kw | 21845 |
| #12 | #7 OR #8 OR #9 OR #10 OR #11 | 59789 |
| #13 | (Platinum-Based Chemotherapy):ti,ab,kw OR (Platinum Chemotherapy):ti,ab,kw OR (Platinum therapy):ti,ab,kw OR (Platinum reactions):ti,ab,kw OR (Platinum-Sensitive):ti,ab,kw | 6977 |
| #14 | (Platinum-resistant):ti,ab,kw OR (Platinum hypersensitivity reactions):ti,ab,kw | 664 |
| #15 | #13 OR #14 | 7087 |
| #16 | #6 AND #12 AND #15 | 206 |

**3.Embase**

| Search number | Query | Results |
| --- | --- | --- |
| #1 | 'ovary tumor'/exp | 167,966 |
| #2 | 'ovarian neoplasms':ab,ti OR 'neoplasm, ovarian':ab,ti OR 'ovarian neoplasm':ab,ti OR 'ovary neoplasms':ab,ti OR 'neoplasm, ovary':ab,ti OR 'neoplasms, ovary':ab,ti OR 'ovary neoplasm':ab,ti OR 'neoplasms, ovarian':ab,ti OR 'ovary cancer':ab,ti OR 'cancer, ovary':ab,ti OR 'cancers, ovary':ab,ti OR 'ovary cancers':ab,ti OR 'ovarian cancer':ab,ti OR 'cancer, ovarian':ab,ti OR 'cancers, ovarian':ab,ti OR 'ovarian cancers':ab,ti OR 'cancer of ovary':ab,ti OR 'cancer of the ovary':ab,ti | 95,195 |
| #3 | 'machine learning'/exp | 297,943 |
| #4 | 'machine learning':ab,ti OR 'deep learning':ab,ti OR 'artificial intelligence':ab,ti OR 'prediction model':ab,ti OR 'transfer learning':ab,ti OR 'random forest':ab,ti OR 'artificial neural network':ab,ti OR ann:ab,ti OR 'support vector machine':ab,ti OR svm:ab,ti OR nomogram:ab,ti OR xgboost:ab,ti OR logistic:ab,ti OR 'decision tree':ab,ti OR 'c index':ab,ti OR roc:ab,ti OR auc:ab,ti OR 'external validation':ab,ti | 970,469 |
| #5 | 'platinum-based chemotherapy':ab,ti OR 'platinum chemotherapy':ab,ti OR 'platinum therapy':ab,ti OR 'platinum reactions':ab,ti OR 'platinum sensitive':ab,ti OR 'platinum hypersensitivity reactions':ab,ti OR 'platinum resistant':ab,ti | 16,274 |
| #6 | #1 OR #2 | 178,681 |
| #7 | #3 OR #4 | 1,153,920 |
| #8 | #5 AND #6 AND #7 | 614 |

**4.Web of science**

| Search number | Query | Results |
| --- | --- | --- |
| #1 | TS=(Ovarian Neoplasms) OR TS=(Neoplasm, Ovarian) OR TS=(Ovarian Neoplasm) OR TS=(Ovary Neoplasms) OR TS=(Neoplasm, Ovary) OR TS=(Neoplasms, Ovary) OR TS=(Ovary Neoplasm) OR TS=(Neoplasms, Ovarian) OR TS=(Ovary Cancer) OR TS=(Cancer, Ovary) OR TS=(Cancers, Ovary) OR TS=(Ovary Cancers) OR TS=(Ovarian Cancer) OR TS=(Cancer, Ovarian) OR TS=(Cancers, Ovarian) OR TS=(Ovarian Cancers) OR TS=(Cancer of Ovary) OR TS=(Cancer of the Ovary) | 136,130 |
| #2 | TS=(machine learning) OR TS=(Deep learning) OR TS=(artificial intelligence) OR TS=(Prediction model) OR TS=(Transfer Learning) OR TS=(random forest) OR TS=(artificial neural network) OR TS=(ANN) OR TS=(Support vector machine) OR TS=(SVM) OR TS=(Nomogram) OR TS=(XGboost) OR TS=(Logistic) OR TS=(Decision tree) OR TS=(c-index) OR TS=(ROC) OR TS=(AUC) OR TS=(External validation) | 2,029,716 |
| #3 | TS=(Platinum-Based Chemotherapy) OR TS=(Platinum Chemotherapy) OR TS=(Platinum therapy) OR TS=(Platinum reactions) OR TS=(Platinum-Sensitive) OR TS=(Platinum-resistant) OR TS=(Platinum hypersensitivity reactions) | 72,519 |
| #4 | #3 AND #2 AND #1 | 609 |
