# Supplementary material for: Predictive Value of Machine Learning for Platinum Chemotherapy Responses in Ovarian Cancer: Systematic Review and Meta-Analysis
Source: J Med Internet Res. 2024 Jan 22;26:e48527. doi: 10.2196/48527 (PMC10845031; doi:10.2196/48527)
Supplement: Multimedia Appendix 3 [file jmir_v26i1e48527_app3.docx]

Table S2 Results of bias risk assessment through PROBAST in 19 included articles.

| Author | Year | Number of Models | Model | Dataset | Research participants | Predictors | Outcomes | Statistical analysis |
| --- | --- | --- | --- | --- | --- | --- | --- | --- |
| C. Lan | 2019 | 3 | LR | Train | H | H | U | H |
| C. Lan | 2019 |  | LR | Train | H | H | U | H |
| C. Lan | 2019 |  | LR | Train | H | H | U | H |
| E Sun Paik | 2017 | 1 | LR | Train | H | H | H | H |
| Haiyue Zhao | 2019 | 4 | SVM | Train | H | H | H | U |
| Haiyue Zhao | 2019 |  | SVM | Train | H | H | H | U |
| Haiyue Zhao | 2019 |  | ANN | Train | H | H | H | U |
| Haiyue Zhao | 2019 |  | ANN | Train | H | H | H | U |
| Hong Zheng | 2021 | 1 | LASSO | Train | L | L | L | H |
| Jesus Gonzalez Bosquet | 2016 | 9 | RF | Train | H | H | H | H |
| Jesus Gonzalez Bosquet | 2016 |  | LASSO | Train | H | H | H | H |
| Jesus Gonzalez Bosquet | 2016 |  | ANN | Train | H | H | H | H |
| Jesus Gonzalez Bosquet | 2016 |  | PAM | Train | H | H | H | H |
| Jesus Gonzalez Bosquet | 2016 |  | DDA | Train | H | H | H | H |
| Jesus Gonzalez Bosquet | 2016 |  | LR | Train | H | H | H | H |
| Jesus Gonzalez Bosquet | 2016 |  | PLR | Train | H | H | H | H |
| Jesus Gonzalez Bosquet | 2016 |  | PLS | Train | H | H | H | H |
| Jesus Gonzalez Bosquet | 2016 |  | RF | Train | H | H | H | H |
| Lanbo Zhao | 2021 | 1 | LR | Train | H | H | H | H |
| Nicholas Brian Shannon | 2021 | 4 | XGBoost | Train | H | H | U | H |
| Nicholas Brian Shannon | 2021 |  | XGBoost | Train | H | H | U | H |
| Nicholas Brian Shannon | 2021 |  | XGBoost | Train | H | H | U | H |
| Nicholas Brian Shannon | 2021 |  | XGBoost | Train | H | H | U | H |
| Ruilin Lei | 2022 | 1 | CNN | Train | H | H | L | H |
| Ruilin Lei | 2022 | 1 | CNN | Train | H | H | L | H |
| Siyu Chen | 2022 | 1 | SVM | Train | H | H | H | H |
| Suhyun Hwangbo | 2021 | 1 | LR | Train | L | L | L | H |
| Tianshui Sun | 2020 | 8 | LR | Train | H | H | H | H |
| Tianshui Sun | 2020 |  | LR | Train | H | H | H | H |
| Tianshui Sun | 2020 |  | LR | Train | H | H | H | H |
| Tianshui Sun | 2020 |  | LR | Train | H | H | H | H |
| Tianshui Sun | 2020 |  | LR | Train | H | H | H | H |
| Tianshui Sun | 2020 |  | LR | Train | H | H | H | H |
| Tianshui Sun | 2020 |  | LR | Train | H | H | H | H |
| Tianshui Sun | 2020 |  | LR | Train | H | H | H | H |
| Xiaoping Yi | 2021 | 1 | SVM | Train | H | H | L | H |
| Yong Han | 2012 | 2 | SPC | Train | H | H | H | U |
| Yong Han | 2012 |  | SPC | Train | H | H | H | U |
| Yongmei Li | 2022 | 1 | LR | Train | H | H | U | H |

Among them, C.Lan 's article contains three LR models, Haiyue Zhao 's article contains two SVM models and two ANN models, Nicholas Brian Shannon 's article contains four XGBoost models, and Tianshui Sun 's article contains eight LR models. Yong Han 's article contains two LR models. Ruilin Lei 's article contains two CNN models.
